# Toxicity of Modified Magnetite-Based Nanocomposites Used for Wastewater Treatment and Evaluated on Zebrafish (*Danio rerio*) Model

**DOI:** 10.3390/nano12030489

**Published:** 2022-01-29

**Authors:** Amaimen Guillén, Yeferzon Ardila, Mabel Juliana Noguera, Ana Lucía Campaña, Miranda Bejarano, Veronica Akle, Johann F. Osma

**Affiliations:** 1 CMUA, Department of Electrical and Electronic Engineering, Universidad de los Andes, Cra. 1E No. 19a-40, Bogotá 111711, Colombia; aa.guillon@uniandes.edu.co (A.G.); mj.noguera10@uniandes.edu.co (M.J.N.); al.campana10@uniandes.edu.co (A.L.C.); 2 Neuroscience and Circadian Rhythms Laboratory, School of Medicine, Universidad de los Andes, Cra 1 No. 18a-10, Bogotá 111711, Colombia; ya.ardila@uniandes.edu.co (Y.A.); sm.bejarano@uniandes.edu.co (M.B.); v.akle@uniandes.edu.co (V.A.)

**Keywords:** magnetic nanocomposites, functionalization, zebrafish, survival, malformation, reproduction, ethology

## Abstract

Magnetite-based nanocomposites are used for biomedical, industrial, and environmental applications. In this study, we evaluated their effects on survival, malformation, reproduction, and behavior in a zebrafish animal model. Nanoparticles were synthesized by chemical coprecipitation and were surface-functionalized with (3-aminopropyl) triethoxysilane (APTES), L-cysteine (Cys), and 3-(triethoxysilyl) propylsuccinic anhydride (CAS). All these nanocomposites were designed for the treatment of wastewater. Zebrafish embryos at 8 h post-fertilization (hpf) and larvae at 4 days post-fertilization (dpf) were exposed to the magnetic nanocomposites Fe_3_O_4_ MNP (magnetite), MNP+APTES, MNP+Cys, MNP+APTES+Cys, and MNP+CAS, at concentrations of 1, 10, 100, and 1000 µg/mL. Zebrafish were observed until 13 dpf, registering daily hatching, survival, and malformations. Behavior was tested at 10 dpf for larvae, and reproduction was analyzed later in adulthood. The results showed that the toxicity of the nanocomposites used were relatively low. Exploratory behavior tests showed no significant changes. Reproduction in adults treated during development was not affected, even at concentrations above the OECD recommendation. Given the slight effects observed so far, these results suggest that nanocomposites at the concentrations evaluated here could be a viable alternative for water remediation because they do not affect the long-term survival and welfare of the animals.

## 1. Introduction

The use of nanocomposites based on different materials has increased widely. Nanocomposites have found applications in different fields such as medical, environmental, cosmetic, electronic, and energy applications, etc. Among these materials, magnetite-based nanoparticles, which have magnetic properties [1,2], are very attractive for various applications [3,4,5,6,7]. The functionalized magnetic nanoparticles are useful in wastewater treatment [8,9,10,11] due to their high ratios of surface area to volume and their magnetic properties which allow for easy recovery and reuse. The functionalization of magnetic nanoparticles is achieved by covering the surface of the nanoparticles by physical or chemical means. This method generates a new compound with a magnetic core which can be coated with an organic or inorganic layer, allowing it to have selective properties when used as an absorbent [10,12]. Although initially used for purposes of seeking to mitigate or solve a problem, there are concerns about the potential impact that these materials may have on the environment. Therefore, considering the increasing proliferation of the use of different nanomaterials, it is necessary to evaluate the possible effects of magnetite-based nanocomposites on biological systems. Aquatic ecosystems, including freshwater and marine ecosystems, receive the majority of waste or pollutants derived from human activities, such as extraction of resources, waste treatment, and over-exploitation in general [13]. Added to all these activities is the accelerated growth of nanomaterials for various uses, whose potential impact on the environment is unknown.

Lately, zebrafish models have been used to assess the possible impact of various molecules in water. This model organism is the aquatic model recommended by the Organisation for Economic Co-operation and Development (OECD) [14]. Zebrafish have characteristics that allow compliance with the fundamental principle of animal research known as the three Rs (3Rs) (i.e., replacement, reduction, and refinement) [15,16,17]. Additionally, it is a suitable model because it is a small and robust fish [18] with low maintenance costs [19]. It has rapid organogenesis, and the embryos are almost transparent, making it possible to examine the development of internal structures. Furthermore, about 70% of zebrafish and human genes are homologous [20] and their physiologies are comparable. For these reasons, zebrafish have been used to study the toxicity and effects of drugs and psychoactive substances on neuronal development and behavior [21]. This is consequently a suitable model for evaluating possible effects produced by magnetite-based nanocomposites.

The magnetite-based nanocomposites evaluated here were designed for the removal of pollutants in wastewater and have been tested on heavy metals such as cadmium [22]. Although the results have been promising, the safety of these nanocomposites in the environment must be guaranteed before their widespread use. Likewise, the toxicity parameters of the magnetite-based nanocomposites presented here have not yet been reported using a zebrafish model.

In this study, we proposed to determine the possible effects on the morphology, survival, behavior, and reproduction of zebrafish subjected to treatments with magnetite-based nanocomposites.

## 2. Materials and Methods

### 2.1. Chemicals

Iron (II) chloride tetrahydrate (98%) (FeCl_2_*4H_2_O), glutaraldehyde (25%), and sodium hydroxide (NaOH) (98%) were obtained from PanReac AppliChem (Spain). Iron (III) chloride hexahydrate (97%) (FeCl_3_*6H_2_O), tetramethylammonium hydroxide (TMAH) (25%), (3-aminopropyl) triethoxysilane (APTES) (98%), and L-cysteine (Cys) (97%) were purchased from Sigma-Aldrich (USA). In addition, 3-(triethoxysilyl) propylsuccinic anhydride (CAS) (95%) was purchased from Shanghai Kayi Chemical Co. (Shanghai, China).

### 2.2. Synthesis and Functionalization of Nanoparticles

Magnetite nanoparticles (MNPs) were synthesized by the chemical coprecipitation method. Iron chloride solution was prepared by stirring 6 mL of 1 M FeCl_2_ and 6 mL of 2 M FeCl_3_ in Milli-Q water at 1500 rpm agitation and 90 °C. Two solutions of 6 mL of 2% *v*/*v* of TMAH and 6 mL of 8 M NaOH were prepared, then added to the mixture with the aid of a 78-8110C Programmable Touch Screen syringe pump (Cole-Parmer^®^, Vernon Hills, IL, USA) at a 12 mL/h flow rate. The resulting MNPs were thoroughly washed and separated using a strong neodymium magnet and Milli-Q water until a pH of 7.0 was reached. Following this, the MNPs were sonicated for 30 min using a Vibra-Cell VCX 750 (Sonics & Materials Inc., Newtown, CT, USA).

APTES, Cys, and CAS were used as functionalization molecules; thus, MNPs and four different surface-modified MNPs were studied: MNP+APTES, MNP+Cys, MNP+APTES+Cys, and MNP+CAS. For MNP functionalization, nanoparticle solutions were buffered with NaOH to reach pH 11 with 50 µL of 2% (*v*/*v*) of TMAH, and then sonicated for 20 min. Surface silanization of MNPs with APTES and CAS was carried out by adding 50 µL of 2% (*v*/*v*) APTES or pure CAS to the corresponding MNP solution and sonicating for 10 min. For Cys functionalization, 50 µL of 0.01% (*w*/*v*) Cys was added. The resulting modified MNPs were thoroughly washed using a neodymium magnet with Milli-Q water until a pH of 7.5 was reached.

A second functionalization layer with Cys on MNP+APTES was produced, to obtain MNP+APTES+Cys. This modification was achieved using 50 µL of 0.01% (*w*/*v*) Cys sonicated for 10 min, and excess reagent was washed with Milli-Q water. Before zebrafish exposure to MNPs, the nanocomposites were washed thoroughly with zebrafish-recirculation-system water (electrical conductivity between 600 and 700 µs/cm and pH 7.4–8.0) until pH 7.5 was reached.

### 2.3. Nanocomposites Characterization

The MNPs size was studied by dynamic light scattering (DLS) using a Zetasizer Nano ZS, (Malvern Panalytical, Malvern, Worcestershire, UK). The sample was prepared by taking the synthesized MNPs and diluting to 3% (*w*/*v*) in 1 mL of Milli-Q water with 100 µL TMAH. The surface modification of MNPs with APTES, CAS, and Cys was analyzed via Fourier-transform infrared spectroscopy (FTIR) using a Bruker ALPHA II FTIR Eco-ATR (Bruker, Germany). Spectra were collected in the range of 4000–500 cm^−1^.

### 2.4. Fish Husbandry, Embryo Collection, and Exposure

Wild-type TABS zebrafish were raised in the facilities of the Laboratory of Neuroscience and Circadian Rhythms, School of Medicine, Universidad de los Andes, Bogotá, Colombia. All procedures with animals were approved by the Institutional Animal Care and Use Committee of Universidad de los Andes (CICUAL in Spanish) with the code C.FUA_19-004. The animals were bred in an automatic recirculation system (Aquaneering Inc., San Diego, CA, USA) with optimal water conditions for the species (28 ± 0.5 °C, electrical conductivity between 600 and 700 µS/cm, pH 7.4–7.6, and a 14:10 h light–dark photoperiod). Animals were fed twice a day with Aquatox Fish Diet rearing food from Zeigler, enriched with live brine shrimp (*A. salina*—INVE Aquaculture, Inc., Salt Lake City, UT, USA). The development of the zebrafish embryo and larvae were observed using an AZ1000M stereoscopic microscope (Nikon, Tokyo, Japan). Zebrafish used for exposure were obtained from spawning adults in crossing tanks.

### 2.5. Treatments

Five types of magnetic nanocomposites were evaluated following the OECD guide for embryonic toxicity tests with additional analysis [14]. Briefly, all nanocomposites were evaluated at concentrations of 1, 10, 100, and 1000 µg/mL. Animals at 8 hpf and 4 dpf were exposed to the nanocomposites for 96 h with treatment changes every 24 h, maintaining the temperature, pH, and conductivity conditions. Groups of 30 animals per concentration were used, divided into three wells in a six-well plate. Egg water was used as a control, and observations were made every day. The experimental time is described in the Supplementary Material, Figure S1.

### 2.6. Mortality, Hatching, and Malformations

During nanocomposite treatment days, observations were made to determine toxicity on embryos and larvae. The mortality of embryos and larvae fish was determined by daily observations of movement, heartbeat, and blood circulation [23] using a stereoscopic microscope (AZ1000M, Nikon, Japan). Embryos were treated at 8 hpf, and their morphological abnormalities were recorded at 24, 48, 72, and 96 h using the stereoscopic microscope, classified according to the OECD protocols into groups such as coagulated embryos, lack of somite formation, non-detachment of the tail, and lack of heartbeat [14]. Hatching was observed between 48 and 120 hpf. A similar procedure was used on the larvae treated at an age of 4 dpf and exposed for 96 h. During the treatment period, the animals were not fed or allowed to have contact with other individuals in the laboratory. Both groups were fed from the ninth day to the end of the observations and euthanized according to the laboratory protocols. Malformations and survival data were taken up to 13 dpf. The malformations were classified as edemas or abnormalities [23,24].

### 2.7. Reproduction Test

Fish exposed to the nanocomposites at 1000 µg/mL for 96 h between 8 hpf and 96 hpf that survived, were later used for evaluation of the effect on their reproduction. They were raised in an independent system for up to 1 year and crossed between themselves. The fertilized embryos were monitored for survival for 13 days. Reproduction crosses were made between treated males and treated females. In addition, crosses of treated females with untreated males, and crosses within groups of untreated animals were performed as reference controls.

### 2.8. Behavioral Tests

Groups of 30 zebrafish larvae at 6 dpf were exposed to each of the five types of magnetic nanocomposites at concentrations of 10 and 100 µg/mL for 96 h. At 10 dpf, animals underwent behavioral testing, including open-field, startle response, and color preference tests, using a DanioVision Observation Chamber and the results were analyzed with EthoVision^®^ XT 14 software (Noldus Information Technology, Wageningen, The Netherlands). Open-field videos were recorded for 5 min, and then the distance traveled, average speed, and freezing time of the animals were analyzed. After that, the startle response time was evaluated (the animal’s reaction time to a single white light flash of 0.1 s). The distance traveled, speed, and elongation during the 5 s before and after the flash of light were measured. Another group of treated animals were studied for the color preference test. For this test, the wells of a 6-well plate were divided into two regions, one green and the other transparent, while the behavior of the larvae in terms of time and frequency of entry into each region was monitored.

### 2.9. Statistical Analysis

Survival, hatching, and malformations were evaluated using two-way ANOVA (with nanocomposite type and concentration as factors), followed by a post hoc Bonferroni test, and were expressed as the mean ± standard error of the mean (SEM). Reproduction data on animals treated with nanocomposites were analyzed using one-way ANOVA followed by a post hoc Tukey test, and were expressed as the mean ± SEM. Behavior data for treated larvae from 6 to 10 dpf were analyzed with one-way ANOVA followed by a post hoc Tukey test, and were expressed as the mean ± SEM. The statistical analyses were developed in Minitab 19 Statistical Software, and the level of significance was defined as *p* ≤ 0.05 for all comparisons.

## 3. Results

### 3.1. MNPs Characterization

**Figure 1:**
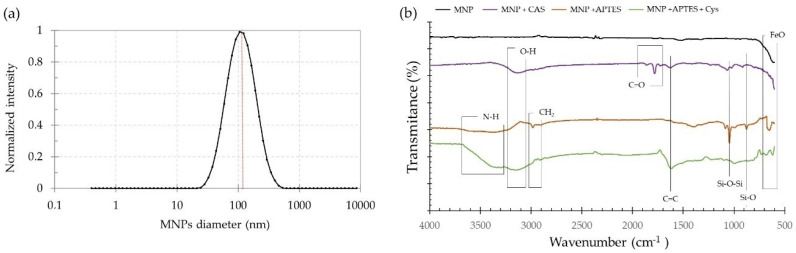
(**a**) DLS result of size distribution of MNPs solution with mean size centered at 125.70 ± 68.05 nm. (**b**) FTIR spectra of MNPs and modified MNPs with CAS, APTES, and APTES+Cys.

MNPs showed a size distribution with a mean particle size of 125.70 ± 68.05 nm. The polydispersity index (PDI) of 0.233 indicated that the synthesized particles exhibited uniform sizes (Figure 1a). Figure 1b shows the IR spectra of the MNPs before and after the surface modification. Peaks near 1100 and 800 cm^−1^ were attributed to Si-O-Si and Si-O stretching vibrations, respectively [25]. These bands were found in the modified MNPs as shown, and they provided further evidence of the correct silanization of the surface. Peaks around 1800 cm^−1^ due to C=O stretching of the anhydride group in CAS can also be seen on MNPs modified with CAS. Peaks around 3500–3000 cm^−1^ and 1620 cm^−1^ can be related to O-H stretching of carboxyl group and C-C stretching, respectively, and could be found in glutaraldehyde and Cys in the MNPs modified with CAS and APTES+Cys. N-H stretching present in APTES and Cys was observed as a broad absorption band around 3200–3600 cm^−1^, which can be attributed to free amine groups after conjugation of these molecules with MNPs. MNPs exhibited adsorption bands around 700–600 cm^−1^, which can be attributed to the Fe-O bond of iron oxide [26]. Weak bands at 2700–3000 cm^−1^ in modified MNP spectra were related to the C-H stretching vibration that is also found in pure APTES, CAS, and Cys. However, the zeta potential of the nanocomposites was expected to be negative for MNP, MNP+Cys, and MNP+CAS in the ranges of −31.9 to −45.9 mV [27,28], −30 to −60 mV [29,30], and −40 mV [31], respectively. For the APTES-functionalized nanocomposites, positive zeta potentials were expected in the range from 29.1 to 47.5 mV [32,33] evaluated at neutral pH values (7.0 to 7.5).

### 3.2. Exposure to Nanocomposites in Embryos

**Figure 2:**
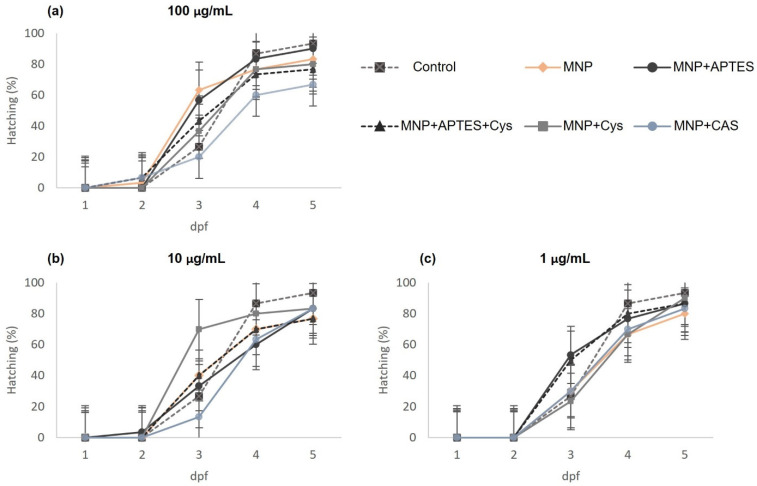
Percentage hatching in embryos treated with nanocomposites compared with the control group. (**a**) Treatments at concentration of 100 µg /mL. (**b**) Treatments at concentration of 10 µg/mL. (**c**) Treatments at concentration of 1 µg/mL. Data are shown as percentage of hatchings ± SEM (*n* = 180 embryos per concentration).

Exposure to magnetic nanocomposites did not alter the embryo hatching rates compared to the control group, and hatching occurred in all groups until the fifth dpf. However, a low hatching rate was observed for the MNP+CAS-treated group at 100 µg/mL, up to 26% less than in the control, as shown in Figure 2a, and 16% less than for the concentrations of 1 and 10 µg/mL in the same nanocomposite group. Embryos from all groups started to hatch after 72 hpf, although there were at least two hatchings in groups other than the control at 2 dpf (MNP, MNP+APTES+Cys, and MNP+CAS at 100 µg/mL, as shown in Figure 2a) and in the MNP+APTES group at 10 µg/mL (Figure 2b). Overall, hatching was dependent on the concentration of the nanocomposites, and averaged 85, 81, and 79% for concentrations of 1, 10, and 100 µg/mL, respectively. Embryos that did not hatch during treatment were classified as coagulated embryos, lack of somite formation, non-detachment of the tail, or lack of heartbeat [23].

**Figure 3:**
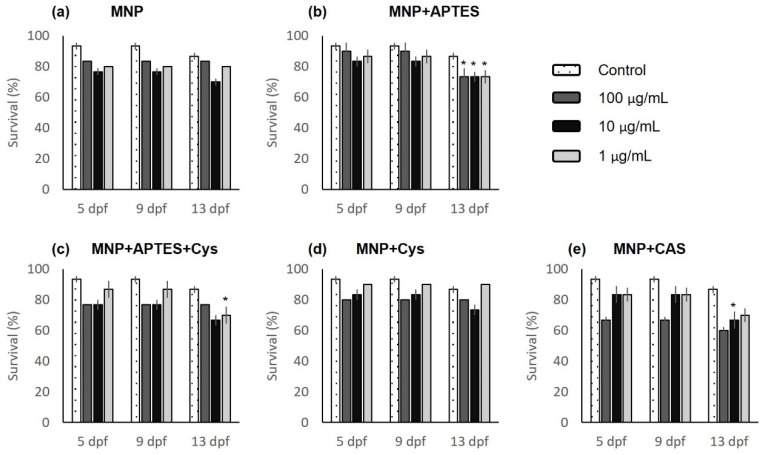
Survival analysis of embryos treated with nanocomposites: (**a**) magnetite nanoparticles (MNPs); (**b**) MNP+APTES; (**c**) MNP+APTES+Cys; (**d**) MNP+Cys; (**e**) MNP+CAS. Data are expressed as the mean ± SEM (*n* = 30). The * represents significant difference *p* ≤ 0.05 in relation to control group.

Survival was evaluated up to 13 dpf, as shown in Figure 3. It can be seen that the control group had the highest survival rates, between 10 and 16% more than the average of the groups treated with nanocomposites, and it is also shown that as the concentration of nanocomposites increased, survival decreased. Survival in the treatment groups was significantly different compared to the control (*p* = 2 × 10^−10^). Embryos treated with MNP+APTES presented, at the end of the observations, the same survival percentage (73%) at the three concentrations evaluated (1, 10, and 100 µg/mL), as shown Figure 3b. Embryos treated with MNP+APTES+Cys and MNP+CAS showed average survival rates of 71 and 66%, respectively; the lowest of all groups, as shown in Figure 3c, e. After the control group, the embryos treated with MNP+Cys had the highest survival rates, with an average of 81%; 5.5% less than the control group, as shown in Figure 3d.

### 3.3. Exposure to Nanocomposites in Larvae

**Figure 4:**
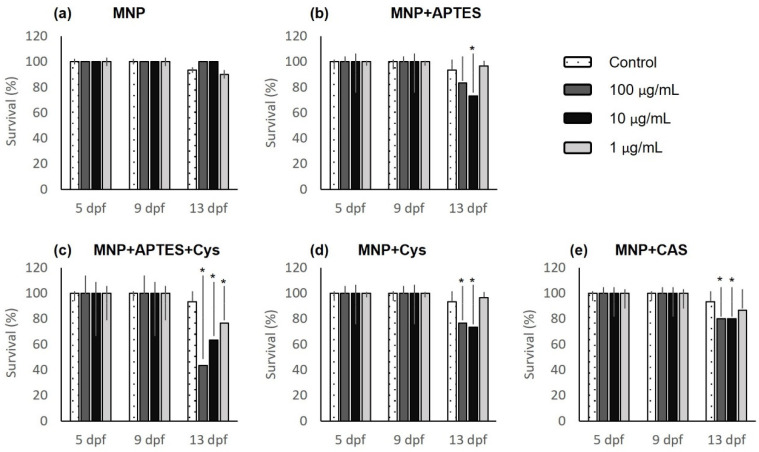
Survival analysis for larvae treated with nanocomposites: (**a**) magnetite nanoparticles (MNPs); (**b**) MNP+APTES; (**c**) MNP+APTES+Cys; (**d**) MNP+Cys; (**e**) MNP+CAS. Data are expressed as the mean ± SEM (*n* = 30). The * represents significant difference *p* ≤ 0.05 in relation to control group.

Statistically different survival rates were observed for animals treated with MNP+APTES+Cys, as shown in Figure 4c with *p* = 0.004. These larvae presented the lowest survival rates up to 13 dpf for each of the concentrations evaluated, at 77, 63, and 43% for concentrations of 1, 10, and 100 µg/mL, respectively. Only survival at 13 dpf was dependent on concentration for this treatment. All exposed larvae survived each treatment from 5 dpf to 8 dpf but started to die after 10 dpf (Figure 4). However, at the end of the observation period (13 dpf), the average survival rate of all the animals treated with nanocomposites was higher than 82%. Only the larvae treated with MNP+APTES+Cys presented an average survival percentage below this value (61%).

**Figure 5:**
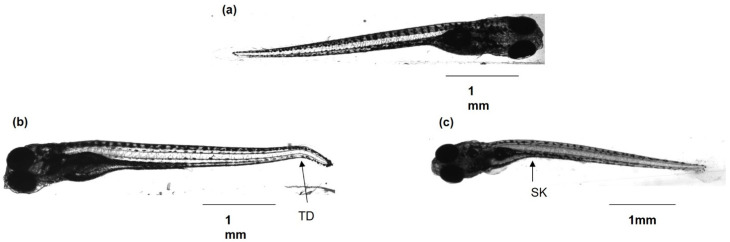
Microscopic representative images of normal and malformed larvae after hatching, which were exposed to different magnetite nanocomposites (magnification: 5×). (**a**) Normally developed larva (7 dpf). (**b**) Larva (10 dpf) treated to 4 dpf with MNPs at 100 µg/mL. (**c**) Larva (10 dpf) treated to 4 dpf with MNP+CAS at 100 µg/mL. Abbreviations: SK: slight kyphosis; TD: tail deformation.

Malformations appeared at 48 h after the beginning of the treatments (6 dpf) for the MNP+CAS group at 100 µg/mL and the MNP+APTES+Cys and MNP+Cys groups at 1 µg/mL. Slight kyphosis was evident on some animals (Figure 5b,c). The group of animals treated with MNP+APTES+Cys was the only one that presented a statistically significant high number of malformations at the three concentrations evaluated (*p* = 1.97 × 10^−10^), with 27% at 1 µg/mL and 33% at 10 and at 100 µg/mL.

### 3.4. Behavior Test

**Figure 6:**
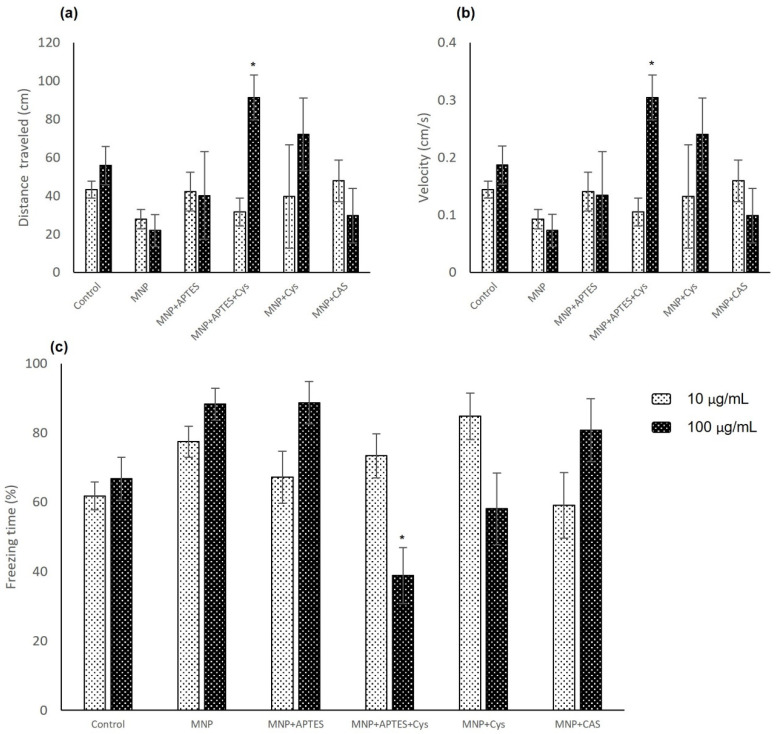
Open-field analysis in larvae that were subjected to nanocomposite treatments at 10 and 100 µg/mL between 6 to 10 dpf: (**a**) distance traveled; (**b**) average speed; (**c**) freezing time. Data are expressed as the mean ± SEM (*n* = 15). The * represents significant difference *p* ≤ 0.05 in relation to control group.

Exposure to magnetic nanocomposites appears to slightly alter the behavior of zebrafish larvae. The shortest distances traveled were observed in MNP-treated animals independently of the concentration. In fact, animals treated with MNP at concentrations of 10 and 100 µg/mL presented 35.6% and 59.7% lower distances traveled compared to the control groups, as shown in Figure 6a and Table 1. However, comparisons between treatment and control groups were not shown to be statistically significant (*p* = 0.113). There was a statistically significant difference only at the concentration of 100 µg/mL, with *p* = 0.0192, for animals treated with MNP+APTES+Cys. This group presented a 63.8% greater distance traveled than the control group. For average speed, the behavior was similar, as shown in Figure 6b and Table 2. A statistically significant difference was presented for the animals treated with MNP+APTES+Cys, with *p* = 0.0196. Freezing time, which is a measure of fear, showed that the animals treated with MNP and MNP+APTES, regardless of the concentration used, presented the highest averages for this measure compared to the control groups, with 6.27% and 4.92% increases, respectively, as shown in Figure 6c. However, when compared by concentration, the difference was not statistically significant at 10 µg/mL but at 100 µg/mL, the animals treated with MNP+APTES+Cys showed a statistically significant difference, with *p* = 4.6 × 10^−5^.

**Figure 7:**
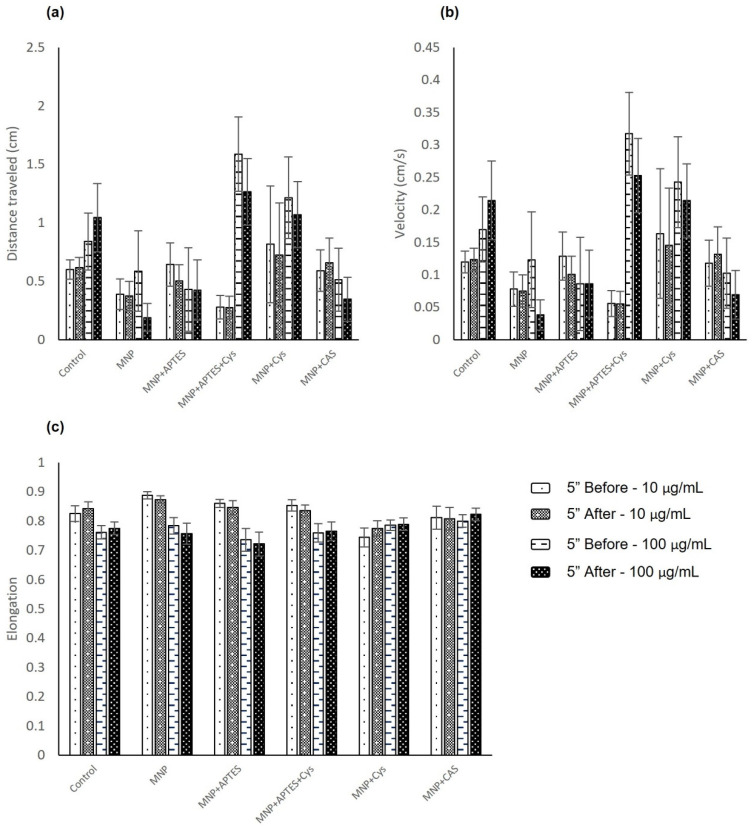
Startle response analysis in larvae that were subjected to nanocomposite treatments at 10 and 100 µg/mL, between 6 to 10 dpf: (**a**) distance traveled; (**b**) average speed; (**c**) elongation. Data are expressed as the mean ± SEM (*n* = 15).

The startle response test evaluated the animal´s reaction time to a single 0.1 s flash of white light, measuring distance traveled, average speed, and body elongation. The analysis was based on the measurement of these variables during the 5 s before and after the light flash. In general, animals treated with nanocomposites showed a decreased distance traveled and speed after the light flash, except those treated with MNP+CAS at the concentration of 10 µg/mL, as shown in Figure 7a,b and Table 3 and Table 4. When compared by concentration, the larvae treated at 10 µg/mL and MNP+Cys showed the highest distance traveled and speed values in relation to the control group, by up to 17.4%. In contrast, larvae treated with MNP+APTES+Cys showed values below those of the control group for the distance traveled and speed variables during the test, by 55.5%. For the concentration of 100 µg/mL, the larvae treated with MNP+APTES+Cys and MNP+Cys presented values of distance traveled and speed above those of the control group after the light flash, by 21.1% and 2.5%, respectively. However, the results showed that the differences between the groups were not statistically significant. Finally, body elongation at concentration of 10 and 100 µg/mL were not statistically different (Figure 7c).

**Figure 8:**
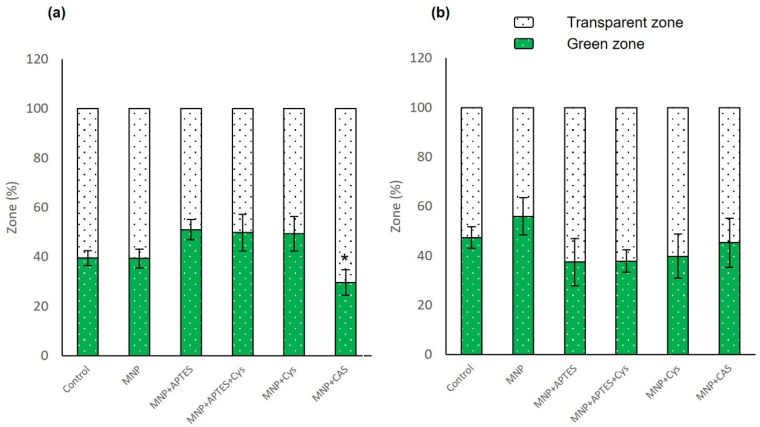
Color preference analysis in larvae that were subjected to nanocomposite treatments between 6 to 10 dpf. (**a**) Animals treated with nanocomposites at concentration of 10 µg/mL and control group. (**b**) Animals treated with nanocomposites at concentration of 100 µg/mL and control group. Data are expressed as the mean ± SEM (*n* = 15). The * represents significant difference *p* ≤ 0.05 in relation to control group.

The color preference test evaluated the behavior of the larvae in terms of time and frequency in each monitored region (green and transparent). Overall, it appears that there were no significant differences between the animals in the nanocomposite treatment groups and the controls. At the concentration of 10 µg/mL, there were statistically significant differences only for the larvae treated with MNP+CAS (*p* = 0.026). For the concentration of 100 µg/mL, larvae treated with MNP were 8.6% above the control group in the green zone, as shown in Figure 8b; however, this was not statistically significant (*p* = 0.478). When the frequency of visits to the different zones was analyzed, animals treated with nanocomposites showed a lower frequency in the number of visits to the green zone compared to the control groups. This shows that there was less movement between zones in the nanocomposite-treated animals.

### 3.5. Reproduction in Animals Treated with Nanocomposites

**Figure 9:**
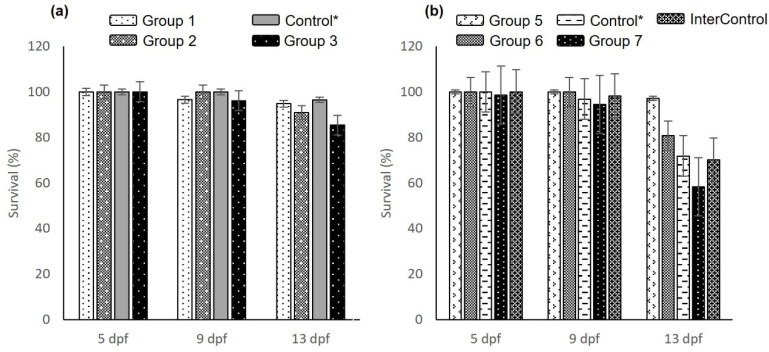
Survival analysis in animals that were subjected to nanocomposite treatments (1000 µg/mL) at embryonic and larval ages. (**a**) Survival of offspring in treated animals evaluated at embryonic and larval ages of 6 months post-treatment. Group 1: females (MNP+APTES+Cys) and males (MNP+Cys); Group 2: females (MNP+APTES+Cys) and males (MNP+CAS); Group 3: females (MNP+Cys) and males (MNP+CAS); Control*: animals that were raised as controls for the experiment under the same conditions as the nanocomposite-treated animals. (**b**) Survival of offspring in treated animals evaluated at embryonic and larval ages of one year post-treatment. Group 5: females (MNP+APTES+Cys) and males (TABS AB); Group 6: females (MNP+Cys) and males (TABS AB); Group 7: females (MNP+APTES+Cys) and males (TABS AB); InterControl: internal control (TABS AB/TABS AB). Data are expressed as the mean ± SEM.

Embryos and larvae subjected to nanocomposite treatment at 1000 µg/mL and raised for reproduction after month six showed an average fertility rate of 40.3%, defined as the number of viable embryos (Nv) at 24 hpf over the total number of eggs collected (Nt), calculated using the formula (Nv*100%)/Nt [34]. The control group raised under the same conditions had an average fertility rate of 33%. At one year, the fertility rates were reduced by an average of 23.7%, while the number of eggs increased by about 57%. Hatching rates of the total eggs collected from the 6-month age group averaged 52.4% in the nanocomposites-treated animals and 31.8% in the control animals, while in the 1-year age group hatching rates were 22.2, 12.7, and 24.8% for the nanocomposites-treated animals, control group (animals that were raised as controls for the experiment under the same conditions as the nanocomposite-treated animals), and internal control group (animals that were raised in an automatic recirculation system), respectively. Surviving larvae were observed until 13 dpf and showed survival values between 85.4 and 94.8% for the 6-month age group in embryos treated with nanocomposites. They were outperformed by the control group by 1.7%, as shown in Figure 9a. For the 1-year age group the survival was between 58.3 and 97.1%, while the control group showed an average of 71.9% and the internal control group presented an average of 70.2%, as shown in Figure 9b. When the groups were compared, there were no significant statistical differences showing that survival was treatment-dependent.

## 4. Discussion

Nanocomposites dissolved in water are currently being evaluated for their effectiveness in the remediation of contaminated water [35,36,37]. Due to the development of this field of nanotechnology as a solution to current environmental problems, it is necessary to have efficient and effective assessment models to examine the impact of nanocomposites on living beings. In the present study, we analyzed the effects of exposure to magnetite-based nanocomposites on zebrafish embryos and larvae, as part of the investigation into the impact of the potential use of nanocomposites on biological systems. We evaluated the effect of nanocomposites on survival, morphology, and reproduction in the short and long term, as well as the behavioral changes of individuals such as locomotion activity, reaction to light stimulus, and color preference. The results of this study are subject to certain limitations. Some of the effects observed during and after exposure to magnetic nanocomposites may be due to alterations in physiological or molecular processes, or in the interactions of individuals, and this was not analyzed in the present study.

The results showed that the nanocomposites evaluated here were not toxic to zebrafish embryos and larvae. Animals treated with magnetite-based nanocomposites showed slight survival, morphology, and behavior variations. The slight changes noted here may be caused not by the magnetite core itself but rather by the functionalizations. One reason may be that the functionalizations cause the new nanocomposites to agglomerate and become unstable in biological or environmental media [38]. It has been found in previous studies that magnetite nanoparticles with and without surface functionalization can change their aggregation state in aqueous media, and this may depend on pH conditions as well as on exposure time [39] and concentration [40]. Although it has been shown that functionalization of magnetite nanoparticles can improve their stability and performance [41], their environmental toxicity still remains unknown. Studies have been undertaken that seek to measure the toxicity of magnetite-based nanoparticles with or without surface coatings. For example, starch-coated nanoparticles were found to be less toxic than bare nanoparticles when gene expression was evaluated in the gills and livers of adult zebrafish [42]. Our results suggest that the exposure time and lack of movement of the nanoparticles during the treatments may cause the observed agglomerations, and this should be further analyzed in future work.

Calculating the magnetite-based nanocomposite concentrations that may present an environmental or health risk might be difficult to achieve precisely. In this study, we have chosen concentrations for toxicity assessment in the range endorsed by the OECD (10 to 100 ug/mL) [14], and in addition, we decided to test the much higher concentration of 1000 µg/mL, in order to be more rigorous. Our results indicated that exposure of zebrafish embryos and larvae to most of the nanocomposites tested here did not affect the survival of the animals. Moreover, as we increased the concentration of the nanocomposites, the survival of the animals in both embryonic and larval stages decreased in different proportions, depending on the type of nanocomposite evaluated. When we tried to fit our data to equation models, we found that our data fitted a second-order polynomial equation model. For example, in embryos, the coefficient of determination R^2^ was 0.6923 for MNP+APTES and 0.9654 for MNP+Cys, which were the extremes of the nanocomposites evaluated. This means that within these ranges of data, we can predict the survival of the treated animals. On the other hand, the most significant changes were observed in embryos and larvae exposed to nanocomposites at 1000 µg/mL. It is worth noting that even at this highest concentration, most of the animals survived to adulthood and bred successfully.

Post-treatment reproduction is a determining factor in analyzing the toxic effect of nanoparticles. However, to the best of our knowledge, post-treatment reproduction tests have not yet been reported in animal models that have been treated with nanocomposites at early ages, for example in embryonic or larval fish. Here, we show that zebrafish treated at embryonic and larval ages with magnetite-based nanocomposites survived to adulthood and had good reproductive rates. In addition, the concentration at which these animals were treated was above the maximum toxicity concentration recommended by the OECD (100 µg/mL) [14], i.e., a concentration of 1000 µg/mL.

To understand the concentrations evaluated here and their use in environmental remediation, we refer to studies that have used magnetite-based nanocomposites for the removal of pollutants in water. In a study on the removal of arsenic (III and V) [43], the authors reported magnetite nanocomposite concentrations for the removal of this contaminant in water of between 0.5 and 2.5 g/L (500 to 2500 µg/mL). However, they showed reusability of up to five cycles and recovery rates of 99% for the nanocomposites [43]. This indicates that even if the highest concentration (2500 µg/mL) was used and 99% of these nanocomposites were recovered, only approximately 25 µg/mL of nanocomposites would remain in the environment (water). Another study on the removal of Pb (II) using magnetite-based nanocomposites [44] evaluated the removal at concentrations of 50 to 400 mg/L (50 to 400 µg/mL). The authors presented reuse of the nanocomposites up to the fifth cycle with recovery percentages close to 85%, indicating that at the highest concentration (400 µg/mL), 60 µg/mL of nanocomposites would remain in the environment. These values are important to highlight since some of the concentrations tested in our study exceeded these values and resulted in no or very low toxicity. This indicates that even if the nanocomposites proposed in this study were used, their environmental impact would be low since they could be recovered from water by taking advantage of the magnetic properties of their core (magnetite) [1,2].

Zebrafish have been used in environmental toxicology to study the effects of nanocomposites including metals, natural metalloids, synthetic compounds, pesticides, pharmaceuticals, and industrial sub-products [45]. Here, we show that zebrafish represent a suitable model to study the elements or compounds that contaminate waterbodies, as well as those that are used to mitigate them. Zebrafish present relevant characteristics for toxicity assessment, such as rapid organ formation, high fecundity rates, and low maintenance costs, compared to other animal models [46]. Zebrafish have been used as a toxicity model at different stages of their development to study the effects associated with different nanomaterials, nanocomposites, or nanoparticles. For example, zebrafish embryos have been used to study the toxicity of silver nanoparticles associated with their shape. In that study, it was determined that the shape of the nanoparticles can affect toxicity in embryos, and the authors concluded that flat nanoparticles were more harmful than spherical ones [47]. Another study used embryos to evaluate hatching rates, survival, heartbeat, and body length of zebrafish subjected to hematite (α-Fe_2_O_3_) nanoparticle treatments. Their results showed that toxicity was slight and was concentration-dependent, i.e., the higher the concentration, the greater the toxicity [48]. The toxicity of iron oxide nanoparticles with different coatings (dextran, chitosan, polyethylene glycol, carboxy-silane, and silica) on zebrafish embryos and larvae has also been reported. The results suggest that toxicity, as measured by hatching rate, malformations, behavior, and survival, was not observed for most of the nanocomposites, except those treated with chitosan, where mortality rates were 100% for concentrations higher than 2 mM (millimolar), thus suggesting further investigation of these types of coatings [49]. Therefore, the zebrafish model is a valid model for evaluating the toxicity of simple or complex nanocomposites such as those evaluated in this study (MNP, MNP+APTES, MNP+APTES+Cys, MNP+Cys, and MNP+CAS).

## 5. Conclusions

In our study, we evaluated the possible effects of magnetic nanocomposites used for the bioremediation of contaminated water on zebrafish embryos and larvae. The nanocomposites evaluated here were not toxic since the results did not show significantly alteration in the survival, morphology, behavior, fertility, or reproduction of the animals subjected to treatment.

Our study demonstrated that exposure to magnetite-based nanocomposites (MNP+CAS, MNP+APTES, and MNP+APTES+Cys) at high concentrations (1000 µg/mL) can induce morphological and physiological alterations in the embryonic and larval stages that could lead to harmful effects, resulting in the reduced long-term survival of the animals (between 2 and 6 ± 1%). However, this concentration of nanocomposites never remains in the environment, since environmental remediation involves the recovery of the nanocomposites and subsequent reuse in different cycles, which has already been demonstrated by other studies that have used magnetite-based nanocomposites for the removal of pollutants in water [43,44].

## Figures and Tables

**Table 1:** Distance traveled (cm) during the open-field test in larvae that were subjected to nanocomposite treatments at 10 and 100 µg/mL between 6 and 10 dpf.

Nanocomposite	Distance Traveled (cm)			
	100 µg/mL	SEM	10 µg/mL	SEM
MNP	21.92	8.38	27.90	5.10
MNP+APTES	40.20	22.85	42.15	10.14
MNP+APTES+Cys	91.45	11.64	31.62	7.27
MNP+Cys	72.17	18.95	39.65	26.98
MNP+CAS	29.73	14.18	47.87	10.88
Control	55.83	10.03	43.30	4.34

**Table 2:** Velocity (cm/s) during the open-field test in larvae that were subjected to nanocomposite treatments at 10 and 100 µg/mL between 6 and 10 dpf.

Nanocomposite	Velocity (cm/s)			
	100 µg/mL	SEM	10 µg/mL	SEM
MNP	0.07	0.02	0.09	0.01
MNP+APTES	0.13	0.07	0.14	0.03
MNP+APTES+Cys	0.30	0.04	0.10	0.02
MNP+Cys	0.24	0.06	0.13	0.09
MNP+CAS	0.09	0.04	0.15	0.03
Control	0.18	0.03	0.14	0.01

**Table 3:** Distance traveled (cm) during the startle response test in larvae that were subjected to nanocomposite treatments at 10 and 100 µg/mL between 6 to 10 dpf.

Nanocomposite	Distance Traveled (cm)							
	5′ Before				5′ After			
	100 µg/mL	SEM	10 µg/mL	SEM	100 µg/mL	SEM	10 µg/mL	SEM
MNP	0.59	0.34	0.39	0.13	0.19	0.11	0.37	0.12
MNP+APTES	0.43	0.36	0.64	0.18	0.42	0.25	0.50	0.14
MNP+APTES+Cys	1.59	0.32	0.28	0.10	1.26	0.28	0.27	0.09
MNP+Cys	1.21	0.35	0.82	0.49	1.07	0.28	0.73	0.44
MNP+CAS	0.51	0.27	0.59	0.18	0.35	0.18	0.66	0.21
Control	0.84	0.24	0.60	0.08	1.04	0.29	0.62	0.09

**Table 4:** Velocity (cm/s) during the startle response test in larvae that were subjected to nanocomposite treatments at 10 and 100 µg/mL between 6 to 10 dpf.

Nanocomposite	Velocity (cm/s)							
	5′ Before				5′ After			
	100 µg/mL	SEM	10 µg/mL	SEM	100 µg/mL	SEM	10 µg/mL	SEM
MNP	0.12	0.07	0.08	0.02	0.04	0.02	0.08	0.02
MNP+APTES	0.09	0.07	0.13	0.03	0.09	0.05	0.10	0.02
MNP+APTES+Cys	0.32	0.06	0.06	0.02	0.25	0.05	0.06	0.02
MNP+Cys	0.24	0.06	0.16	0.09	0.21	0.05	0.15	0.08
MNP+CAS	0.10	0.05	0.12	0.03	0.07	0.03	0.13	0.04
Control	0.17	0.05	0.12	0.02	0.21	0.06	0.12	0.02

## Data Availability

The data and contributions presented in the study are included in the article. Further inquiries can be directed to the corresponding author.

## Supplementary Materials

The following supporting information can be downloaded at: https://www.mdpi.com/article/10.3390/nano12030489/s1. Figure S1: Representation of experimental timeline. Notes: hpf: hours postfertilization, dpf: days postfertilization and mpf: months postfertilization. * After behavioral testing, these larvae were euthanized.
