# Supplementary material for: Toxicity of Modified Magnetite-Based Nanocomposites Used for Wastewater Treatment and Evaluated on Zebrafish (Danio rerio) Model
Source: Nanomaterials (Basel). 2022 Jan 29;12(3):489. doi: 10.3390/nano12030489 (PMC8839930; doi:10.3390/nano12030489)
Supplement: Supplementary file 1 [file nanomaterials-12-00489-s001.zip › nanomaterials-1520647-supplementary.pdf]

nanomaterials

Supplementary Materials

Toxicity of Modified Magnetite-Based Nanocomposites Used for Wastewater Treatment and Evaluated on Zebrafish (*Danio rerio*) Model

Amaimen Guillén [1,2], Yeferzon Ardila [2], Mabel Juliana Noguera [1], Ana Lucía Campaña [1], Miranda Bejarano [2], Veronica Akle [2] and Johann F. Osma [1,*]

[1] CMUA, Department of Electrical and Electronic Engineering, Universidad de los Andes, Cra. 1E No. 19a-40, Bogotá, DC 111711, Colombia; aa.guillon@uniandes.edu.co (A.G.); mj.noguera10@uniandes.edu.co (M.J.N.); al.campana10@uniandes.edu.co (A.L.C.)
[2] Neuroscience and Circadian Rhythms Laboratory, School of Medicine, Universidad de los Andes, Cra 1 No. 18a-10, Bogotá, DC 111711, Colombia; ya.ardila@uniandes.edu.co (Y.A.); sm.bejarano@uniandes.edu.co (M.B.); v.akle@uniandes.edu.co (V.A.)
* Correspondence: jf.osma43@uniandes.edu.co; Tel.: +57-601-339-4949

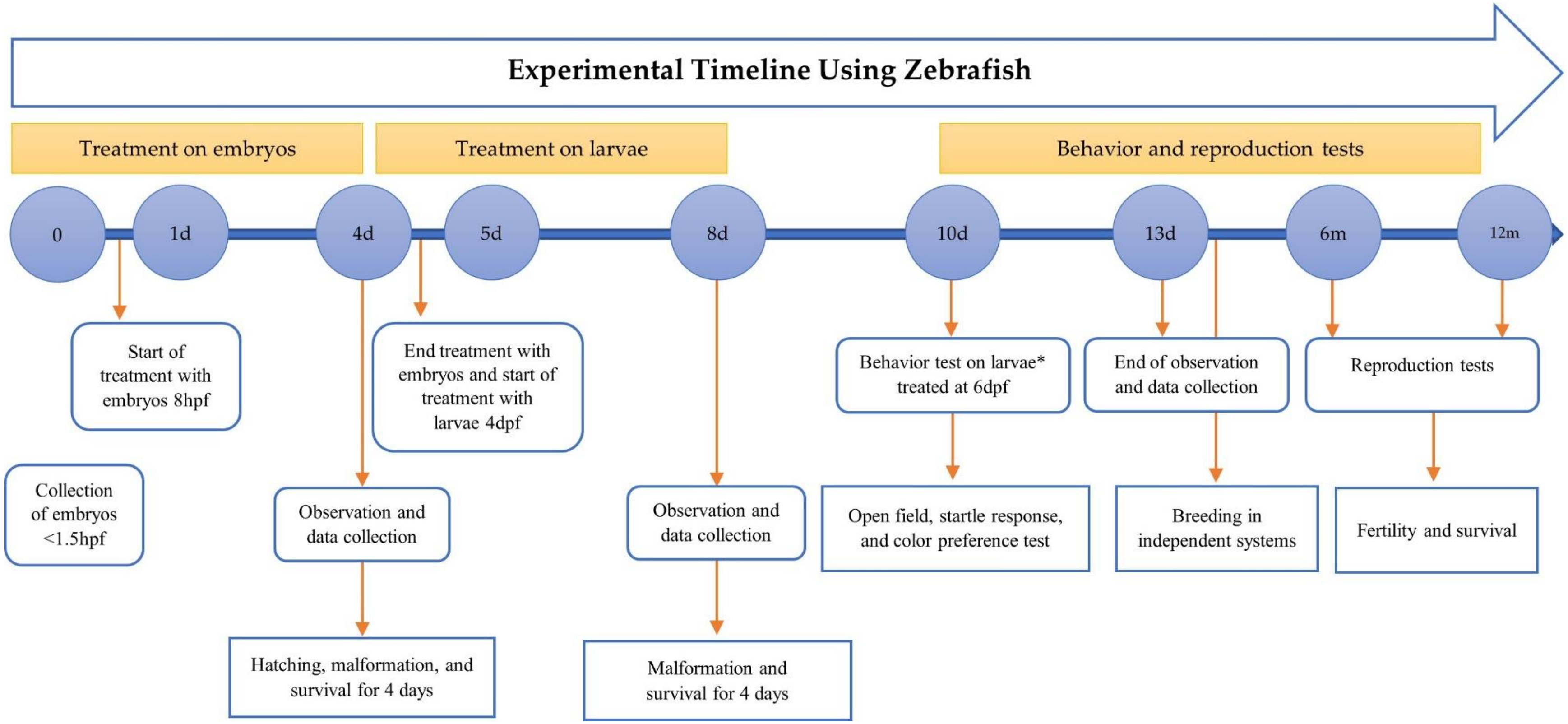

Figure 1. Representation of experimental timeline. Notes (hpf: hours postfertilization, dpf: days postfertilization and mpf: months postfertilization). *After behavioral testing, these larvae are euthanized.
